# COMPETENCY DEVELOPMENT FOR A PROGRAM TO SUPPORT CAREGIVERS OF OLDER ADULTS WITH DEMENTIA: A MODIFIED E-DELPHI METHOD

**DOI:** 10.1093/geroni/igac059.907

**Published:** 2022-12-20

**Authors:** Madison Huggins, Gloria Puurveen, Barb Pesut

**Affiliations:** UBC, Kelowna, British Columbia, Canada; UBC, Kelowna, British Columbia, Canada; UBC, Kelowna, British Columbia, Canada

## Abstract

Nav-CARE is a program that utilizes volunteer navigators to support older adults with life-limiting illnesses who are living in the community. Currently, Nav-CARE is being adapted and expanded to support family and/or friend caregivers of older adults with dementia. In order to begin the process of adapting this program a modified e-Delphi method was utilized. This method consisted of presenting three sequential questionnaires to an expert panel (n=50) of individuals with knowledge of and/or experience in caregiving, dementia, volunteerism and/or navigation. Consensus was established regarding the importance of various caregivers’ needs and the competencies volunteer navigators require in order to meet these needs. This process resulted in a final list of 46 caregivers' needs and 41 volunteer navigator competencies. Two key findings suggest that there is a crucial need for increased access to respite, which influences the caregivers' ability to benefit from additional supports, and that dementia stigma is prominent, which negatively impacts the caregiving experience. Additional findings highlight the need for increased knowledge of dementia among providers, caregivers, and community members, the importance of establishing strong volunteer navigator-caregiver relationships, and the need to balance the agency of older adults living with dementia and their caregivers. Findings from this study informed the development of training curriculum for volunteer navigators, and will be used to guide the ongoing development of the adapted Nav-CARE program.

